# 102. Assessing ChatGPT Performance in the Brazilian Infectious Disease Specialist Certification Examination

**DOI:** 10.1093/ofid/ofad500.018

**Published:** 2023-11-27

**Authors:** Alexandre Chaves Fernandes, Maria Eduarda Varela Cavalcanti Souto, Thais Barros Felippe Jabour, Kleber G Luz, Eveline Pipolo Milan

**Affiliations:** Federal University of Rio Grande do Norte, Natal, Rio Grande do Norte, Brazil; State University of Rio Grande do Norte, Natal, Rio Grande do Norte, Brazil; Federal University of Rio Grande do Norte, Natal, Rio Grande do Norte, Brazil; Federal University of Rio Grande do Norte, Natal, Rio Grande do Norte, Brazil; Federal University of Rio Grande do Norte, Natal, Rio Grande do Norte, Brazil

## Abstract

**Background:**

Advances in artificial intelligence have the potential to impact medical fields, including the use of natural language processing-based models, such as ChatGPT. The ability of the ChatGPT to provide insightful responses across diverse fields of expertise could assist in medical decision-making and knowledge management processes. ChatGPT has already demonstrated high accuracy in medical examinations such as the USMLE. To explore the potential of this tool in various contexts, our study aimed to evaluate the accuracy of the ChatGPT in the 2022 Brazilian Infectious Disease Specialist Certification Examination.

**Methods:**

We conducted a test to evaluate the performance of GPT-3.5 and GPT-4 on the 2022 Brazilian Infectious Disease Specialist Certification Exam. A theoretical exam, consisting of 80 multiple-choice questions with five alternatives, was used to test performance. The GPT was given a command containing the question statement and alternatives, and a brief comment on the logic behind the answer was requested. Descriptive statistics were used to analyze the absolute performance of the correct answers in the ChatGPT-3.5 and ChatGPT-4 models. In addition, the degree of correlation between answers and performance throughout the test was estimated using Spearman's coefficient and a logistic regression curve, respectively.

**Results:**

Of the 80 questions in the exam, four were excluded because they were invalidated in the final answer key. ChatGPT-3.5 had an accuracy of 53.95% (41/76), whereas ChatGPT-4 had an accuracy of 73.68% (56/76). Spearman's correlation coefficient between the two models was 0.585. There was a slight trend towards improvement in ChatGPT-4 performance throughout the test, as observed in the logistic regression curve.

Comparison of Accuracy between ChatGPT 3.5 and ChatGPT4

**Figure:**
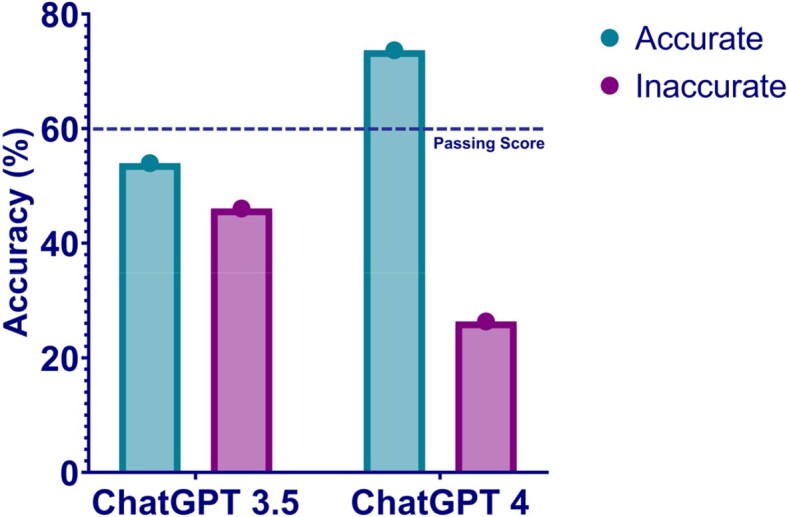

The graph shows the percentage of accuracy for the two GPT models. The performance of ChatGPT-4 was superior to ChatGPT-3.5.

Distribution of Correct and Incorrect Responses by ChatGPT-4 in Medical Test Questions

**Figure:**
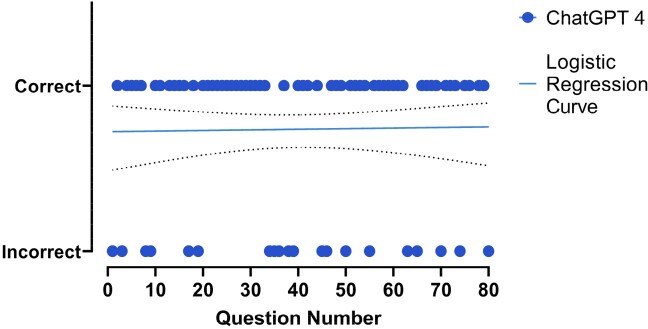

The graph displays the distribution of responses generated by ChatGPT-4. The logistic regression curve shows a slight upward trend, indicating a slight improvement in performance as the questions were answered.

**Conclusion:**

ChatGPT-4 achieved performance above the 60% minimum threshold required for the certification exam. This indicates that it is a promising technology in various fields, including infectious diseases. However, its potential applications and associated ethical dilemmas must be thoroughly assessed. This advancement also highlights the need for medical education to concentrate on developing competence, skills, and critical thinking rather than relying solely on memorization

**Disclosures:**

**All Authors**: No reported disclosures

